# Pulmonary Artery Aneurysm as a Cause of Massive Hemoptysis: Diagnosis and Management

**DOI:** 10.1155/2011/141563

**Published:** 2011-06-30

**Authors:** Peter Corr

**Affiliations:** Department of Radiology, United Arab Emirates University, P.O. Box 17666, Al Ain, UAE

## Abstract

Massive hemoptysis is a life-threatening medical emergency. Prompt radiological diagnosis and management are essential. I present a case of an inflammatory pulmonary aneurysm (Rasmussen aneurysm) from active pulmonary tuberculosis. This is an uncommon cause for massive hemoptysis which was successfully treated by endovascular coiling.

## 1. Introduction

Massive hemoptysis is defined as the expectoration of more than 30 mls of blood within a 24-hour period. This is a life-threatening medical emergency with a mortality of 50–80% if untreated [1]. Destructive lung diseases from many causes lead to massive hemoptysis from neoplasms, tuberculosis, bronchiectasis, and cystic fibrosis [2]. Almost all cases of hemorrhage originate from bleeding hypertrophied bronchial arteries that can usually be occluded using endovascular techniques [3]. Bleeding into cavities from tuberculosis is common, but massive hemoptysis is rare and has been described as a result of pseudoaneurysms of the pulmonary artery where the artery is focally weakened by the inflammatory infiltrate.

## 2. Case Report

**Figure 1:**
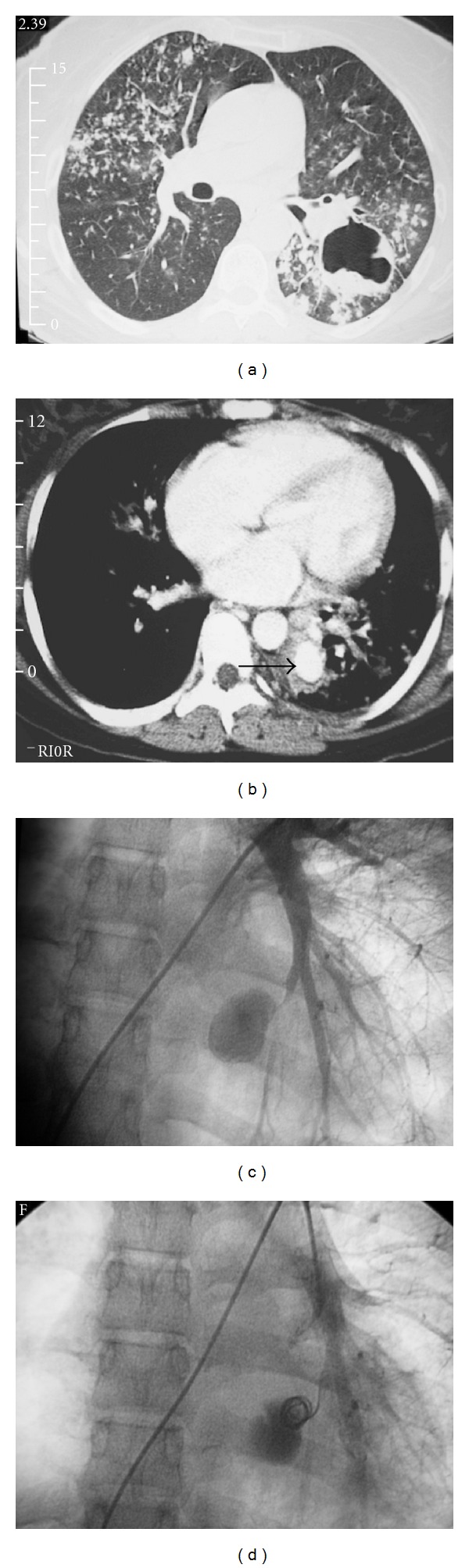
(a) Lung CT demonstrates bilateral nodular infiltrates with a left lower lobe cavity containing hematoma from active pulmonary tuberculosis. (b) Contrast CT demonstrates focal contrast enhancement in the left lower lobe pulmonary aneurysm (arrow). (c) Selective left lower pulmonary arteriogram demonstrates the saccular Rasmussen aneurysm with a narrow neck. (d) Post embolization pulmonary angiogram demonstrates occlusion of the neck of the aneurysm with a single steel coil with residual contrast within the aneurysm.

A 23-year-old women present to the emergency room with a one day history of coughing up five cups filled with blood. She complained of a chronic cough for 3 months, loss of weight, and night sweats. Clinical examination detected that the patient was diaphoretic and distressed. She was anemic with a hemoglobin of 6 g/dL and hypotensive with a blood pressure of 100/60 mmHg. The patient was resuscitated and was transfused with 4 units of packed red blood cells to a hemoglobin level of 10 g/dL. Chest X-ray revealed active pulmonary tuberculosis with multiple cavities in both lungs. Sputum was positive for acid-fast bacilli. A CT scan of the lungs confirmed the diagnosis (Figure 1(a)). Focal contrast enhancement in the left lower lobe parenchyma was detected on the contrast CT scan (Figure 1(b)). A pulmonary aneurysm in the left lower lobe was suspected. Once the patient was fully resuscitated, selective bronchial arteriography and pulmonary angiography were performed. A 5 cm diameter oval aneurysm was detected originating from the left lower lobe posterior-medial segment pulmonary artery (Figure 1(c)). The bronchial arteries were not enlarged, and no active contrast extravasation was detected from them. A single Gianturco steel coil 5 mm in diameter was selectively placed at the neck of the aneurysm with occlusion of the pulmonary aneurysm (Figure 1(d)). The patient's hemoptysis resolved over the next 12 hours. The patient was treated with a full course of TB therapy and made a good recovery.

## 3. Discussion

Inflammatory pulmonary artery aneurysms are an extremely uncommon cause of hemoptysis in pulmonary tuberculosis. Far more commonly, hemorrhage occurs from hypertrophied bronchial arteries [3]. Fritz Waldemar Rasmussen, a Danish physician, first described 11 cases of pulmonary aneurysms in patients with tuberculosis in 1868 [4]. Auerbach in 1939 detected pulmonary aneurysms in 4% of patients at autopsy dying from tuberculosis [5]. In this era of multidetector CT, the prevalence of pulmonary aneurysms detected in a large retrospective series of 189 patients with massive hemoptysis from tuberculosis was 6.9% [5]. Careful evaluation of a pre- and postcontrast CT scan of lungs will demonstrate focal contrast enhancement within the pulmonary aneurysm [6]. This may be the first clue to the correct cause of the hemoptysis. Endovascular occlusion of the neck of the pulmonary aneurysm is well described in the literature and is usually successful in treating the hemoptysis [7]. Steel coils are the occlusive material of choice although thrombin and cyanoacrylate injection percutaneously under fluoroscopic and ultrasound guidance has been used where vascular access is not possible [7, 8].

Although uncommon, Rasmussen pulmonary aneurysms are an important cause for massive hemoptysis in patients with tuberculosis. Careful evaluation of the post contrast CT study may be helpful in suggesting the correct diagnosis.
